# Prostate Cancer: Is It a Battle Lost to Age?

**DOI:** 10.3390/geriatrics1040027

**Published:** 2016-11-03

**Authors:** Venkatesh Vaidyanathan, Nishi Karunasinghe, Anower Jabed, Radha Pallati, Chi Hsiu-Juei Kao, Alice Wang, Gareth Marlow, Lynnette R. Ferguson

**Affiliations:** 1 Discipline of Nutrition and Dietetics, FM & HS, University of Auckland, Auckland 1023, New Zealand; rpal628@aucklanduni.ac.nz (R.P.); b.kao@auckland.ac.nz (C.H.-J.K.); alice.wang@auckland.ac.nz (A.W.); l.ferguson@auckland.ac.nz (L.R.F.); 2 Auckland Cancer Society Research Centre, Auckland 1023, New Zealand; n.karunasinghe@auckland.ac.nz; 3 Department of Molecular Medicine and Pathology, FM & HS, University of Auckland, Auckland 1023, New Zealand; a.jabed@auckland.ac.nz; 4 Experimental Cancer Medicine Centre, Cardiff University, Cardiff CF14 4XN, UK; MarlowG@cardiff.ac.uk

**Keywords:** prostate cancer, aging, single nucleotide polymorphism, epigenetics, miRNA, CRISPR/Cas9

## Abstract

Age is often considered an important non-modifiable risk factor for a number of diseases, including prostate cancer. Some prominent risk factors of prostate cancer include familial history, ethnicity and age. In this review, various genetic and physiological characteristics affected due to advancing age will be analysed and correlated with their direct effect on prostate cancer.

## 1. Introduction

Aging is the irreversible process by which individuals undergo various physiological changes, and become vulnerable to various diseases, which in some cases can also be the cause of mortality [1,2]. Age is often considered an important non-modifiable risk factor for a number of diseases which include, but are not limited to neurodegenerative diseases such as age-specific Alzheimer’s disease [3]; cardiovascular diseases such as coronary heart disease, stroke [4,5], and hypertension [6]; and certain cancers [7,8] including prostate cancer (PCa) [9,10].

PCa has one of the highest incidence rates amongst all diagnosed cancers in males worldwide [11]. According to 2011 statistics, in males, PCa was the most common cancer registration in New Zealand, making up 27.3% of all male cancer registrations, and is a significant burden to the Public Health System [12]. Some prominent risk factors of PCa include familial history, ethnicity and age [13]. Only a very few cases of PCa are diagnosed in men less than 50 years old and around three-quarters of all the cases diagnosed are in men 65 years or older. For this review, we are considering 65 years as the cut-off [14]. Certain other factors which can also be related to PCa as risk factors are reproductive hormonal imbalances [12] and lifestyle factors such as, diet and smoking tobacco [12,13].

**Figure 1:**
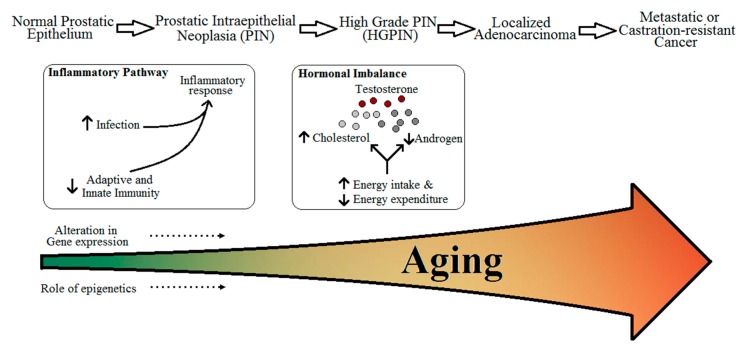
Relation between aging and risk and progression of prostate cancer.

In this review, we will be looking into four parameters as risk for PCa which can be affected due to aging (Figure 1):

(i) Immunity and inflammatory response,
(ii) Cholesterol metabolism and obesity,
(iii) Free testosterone levels,
(iv) Genetic and epigenetic effects.

We will then correlate these changes with advancing age and try to establish if they have a direct effect on the onset and progression of PCa or not. By the end of this review we aim to answer three important questions with regards to the diagnosis and progression of PCa:

1. Is PCa a gerontological disorder fuelled by an aging immune system, altered cellular metabolism, decreased levels of testosterone, and alterations in gene expression?
2. Is there potential for slowing or reverting these changes?
3. If the answer to question 1 is ‘yes’, can diagnostic tools distinguish aggressive PCa from non-aggressive PCa for possible early interventions?

To our knowledge, this is the first conclusive review to look into the alterations in risk factors for PCa with progressing age as a standard of variability in patients.

## 2. Risk Factors for Prostate Cancer

Most cancers are defined as complex diseases involving both environmental and genetic determinants as risk factors [15]. It is well documented that cancers of almost all the organs and organ systems can be caused due to environmental and lifestyle factors, including, and not restricted to, smoking tobacco, alcohol consumption, infectious agents, occupation, diet and obesity [16]. We suggest that the entire span of life spent pursuing the various lifestyle habits may have an accumulated effect on various factors such as immunity, inflammation, and even expression of the genes. Therefore the role of aging is of much importance not only to understand the role it plays directly as a risk for diseases, but also indirectly. 

With regards to PCa, it is well-established that there are three major risk factors. These are advancing age, ethnicity, and familial history [17]. Recent studies point out that alterations in genetic and epigenetic make-up are the basis for the development of malignancies [18]. 

For most cancers, including PCa, however, the assessment of the exposures to external (or, environmental factors) and their effects still remains a challenge. Our understanding of the etiology of cancer in terms of environmental factors and genetic susceptibility is still rather limited, and the interplay among these etiological constituents is poorly understood [15]. For this review, as previously mentioned, we are focusing on the progressing age and its effect on the expression and progression of PCa.

## 3. Aging, Immunity, Inflammatory Response, and Prostate Cancer 

Immune defense in higher vertebrates functions by the detection of a wide range of molecular patterns which are foreign to the tissues, inducing innate immunity and an inflammatory response [19,20]. The elderly exhibit an increase in susceptibility to various autoimmune, inflammatory, and/or infectious diseases [20,21]. Immunosenescence, or age-related alterations impairing the proper functioning of the immune system, is considered to be the major cause of most of the diseases associated with old age [20]. Immunosenescence has an impact on both adaptive and innate immunity [22,23].

**Figure 2:**
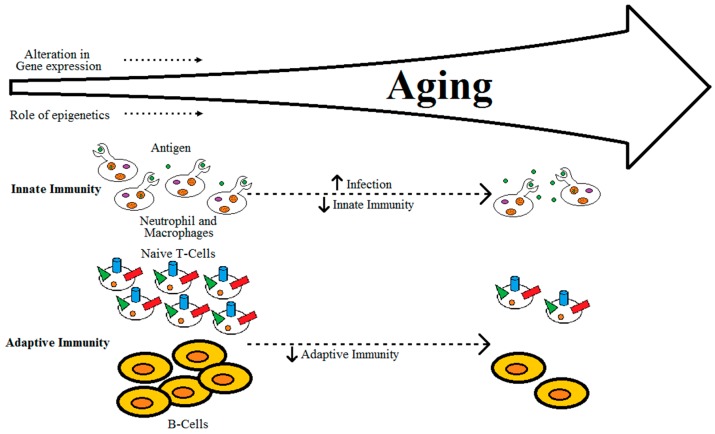
Relation between aging and immunity.

It is well-established that adaptive immunity declines with progressing age, due to factors such as reduced production of naïve T-cells, reduced diversity of antigen-recognition repertoire, alterations in signal transduction in T-cells with changes in the cytokine induction patterns, and reduction in expansion of clonal and functional specific T- and B-cells, as shown in Figure 2 [23,24]. Aging of the thymus can also be linked to decreases in the production of naïve T-cells [23]. Alterations in innate immunity alone due to aging may not be the cause of immunodeficiency in individuals, but lead to dysregulation of immune response [23]. This dysregulation of immune response in aging individuals can eventually lead to either loss or gain of immune activity [20,21,22,23,25]. The gradual decrease of naïve T-cells, accumulation of memory T-cells and the effector CD8+CD28- T-cells, which are instrumental in over-secretion of pro-inflammatory cytokines, can lead to an imbalance in the pro- and anti-inflammatory networks [24,26,27].

Inflammation can have a wide-spread effect on cancer, from development and progression of tumours, to response to therapies [28]. Chronic inflammatory responses induced by biological, chemical, physical, and/or mechanical injuries have been associated with a higher incidence rate of cancer in a wide range of human tissues [28,29]. Inflammation can be linked to cancer through two broad pathways, intrinsic and extrinsic [30,31]. The intrinsic pathway of inflammatory response to cancer is initiated when oncogene activation is induced in the transformed cells, such that activation of transcription factor nuclear factor kappa-light-chain-enhancer of activated B-cells is affected by the production of inflammatory mediators [31]. The extrinsic inflammatory response pathway caused due to infectious conditions also affects the risk of cancer development [31]. Various risk factors, including environmental aspects are also identified to be associated with some kind of chronic inflammation [28]. 

Some of the genes which play a common role as risk factors for PCa and inflammation pathways, include *RNase L* (*RNASEL*), *Macrophage Scavenger Receptor 1* (*MSR1*), *Glutathione S-Transferase P* (*GSTP1*), *Growth Differentiation Factor 15* (*GDF15*), *Toll-like Receptor 4* (*TLR4*), *Macrophage Inhibitory Cytokine-1 (MIC-1)*, *Interleukin 1 Receptor Antagonist* (*IL1RN*), *Interleukin-8* (*IL8*), and *Interleukin-10* (*IL10*) [32]. Kazma, et al. (2015) investigated the association of 320 single nucleotide polymorphisms in 46 genes involved with the inflammation pathway with the risk of PCa in 494 patients with advanced diseases and 536 healthy men of African American and Caucasian ethnicities [33]. Their results indicated that, although innate immunity and the inflammation pathway do not play a central role in the progression of PCa, they may play a role in the overall risk of the disease [33]. Anatomically, since the prostate gland is broadly classified into four lobes, some researchers have also looked into the expression and spread of cancer based on the individual lobe.

Das et al. (2013), while working on changes in proteomic profiles in the different lobes of male rats in different aging stages, have identified that aging in the dorsolateral and ventral lobes affected many proteins that are involved in vital cellular activities, especially inflammatory response [34]. Of the many proteins they identified to be affected by aging, in each lobe, the expression of three proteins was identified to have increased, α-1 inhibitor 3, cysteine and glycine-rich protein, and ANXA1 (by the gene *Annexin A1*). Three proteins have also decreased expression uniformly in both lobes- hypoxia up-regulated protein 1, prolyl-1-hydroxyl-β peptide, and protein disulphide isomerase family A, member 3 [34]. These findings are interesting, as the role of genes is much more evident than the ageing of the organs as such. 

We suggest that aging leads to a decline in immune response, and triggers the inflammatory pathways leading to development of PCa and these factors may not be playing a role in the progression of this disease but the risk of development of disease.

## 4. Aging, Cholesterol Metabolism, and Prostate Cancer

Effects of high fat diets (HFDs) on cholesterol metabolism have been associated with hyperlipidemia in humans [35]. HFDs increase the total and low-density lipoprotein (LDL) cholesterol levels in plasma, decrease high-density lipoprotein (HDL), and increase the total cholesterol to HDL ratio [35]. A life-long consumption of food rich in calories such as red meat, processed food substances (such as meat, dairy and fruit products) and/or high temperature cooking methods [36] increases the chances of obesity and concentrations of triglycerides and cholesterol in serum. This may be due to increased energy intake compared to expenditure leading to a number of diseases such as cancer [16,36]. High cholesterol in circulation has also been considered as a risk factor for solid malignancies, predominantly due to the upregulation of pathways such as cholesterol synthesis and/or inflammatory response [37].

Although cancers originating in different tissues may vary hugely in terms of overall type and etiology, they can still have common attributes of metabolic anomalies [38]. Cancer, at the cellular level, is a disease defined by uncontrolled cell growth and proliferation requiring cellular building blocks such as nucleic acids, proteins, and lipids [38]. Alteration in metabolism in cancer cells permits them to accumulate higher quantities of metabolic intermediates which can be used as building blocks in the body [38]. Detection of cholesterol deposits in tumour cells has made it vital to analyse the cellular function of cholesterol and fatty acid metabolisms leading to the uncontrolled growth in these cells [38,39,40]. Due to the uniqueness of the prostate gland, the association between cholesterol deposits and PCa has been identified to be very strong [39,41]. Oxidation of fatty acids is also the source for an increase in the production of mitochondrial reactive oxygen species (ROS), which at high levels can be harmful to organelles, including the mitochondria [42,43], and various pathological states including cancers such as PCa [44].

**Figure 3:**
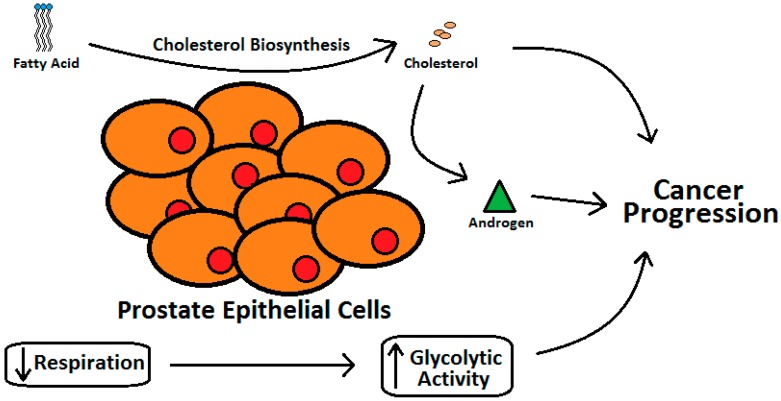
Relation between cholesterol metabolism and progression of prostate cancer.

Various case-control [45,46] and epidemiology studies [47] have demonstrated that individuals with elevated blood cholesterol levels run a higher risk of PCa [40]. The epithelial cells of the prostate gland have two very unique features [41]. Prostate gland cells, owing to higher cholesterol synthesis as compared with the liver cells, have higher cholesterol levels than other tissues in the vicinity and this increase with aging and progression of PCa (Figure 3) [39]. Prostate epithelial cells also express higher level of glycolytic activity with reduced respiration [41,48]. 

It is an inevitable fact that, when subjects undergo studies designed to identify the impact of amount and/or type of fat intake, there is an overall effect on the protein and/or carbohydrate in the diet as well [49]. Due to this, it is extremely difficult to pinpoint the effect that changes in diet have on the well-being of an individual [49]. It is therefore very important to look into details such as genes involved in metabolism to identify the regulation of fat break-down and absorption. The effect of aging on these genes becomes crucial too, as aging not only means that the individuals have had a life-time of certain dietary habits (which may cause higher oxidative stress and/or DNA damage), but also factors such as telomere shortening which leads to senescence [50].

## 5. Aging, Testosterone Level and Prostate Cancer

It is well established that the various symptoms of aging in males include and are not limited to fatigue, reduction in physical strength, lack of energy, lower and eventually loss of libido, reduced sexual performance, depression, and mood swings [51]. Various bodily compositions in healthy males also change with age-associated decline in free and bioavailable testosterone [52]. Some examples of alteration in body compositions include an increase in fat mass and decrease in muscle mass and bone mineral density [53,54,55,56]. 

With testosterone being the primary androgen receptor-activating hormone identified in the Wolffian duct, and also responsible for the development of the primary sexual characteristics [57], it is the main hormone of interest in this review. The overall level of the biochemical testosterone also decreases with aging [51]. What is interesting from the point of research is that the symptomatic evidence suggests normal aging in males to be very similar to those with mild androgen deficiency [51]. In concordance with the core idea of this review, aging in males and the effect on testosterone levels in males with PCa is another important aspect. 

There is a substantial amount of epidemiological data to prove that serum free and total testosterone, and adrenal steroid dehydroepiandrosterone (DHEA) levels decrease with the normal aging process after peaking reaching the age bracket of 20–30 years [58,59,60]. Examples of the decrease in the levels of serum testosterone with aging have been cited in cross-sectional and longitudinal studies [58,61]. On the contrary, certain other androgen axis products such as luteinizing hormone, follicular stimulating hormone and dihydrotestosterone levels, and sex hormone-binding globulin levels have been identified to increase with age [59,62]. Reduction of testosterone could also be at least partly due to it’s being aromatized to estrogen with increasing age [62]. According to Bélanger, et al. (1994), a decline in adrenal DHEA is responsible for up to a 50% reduction in total androgens in men beyond the age of 40 years [63].

**Figure 4:**
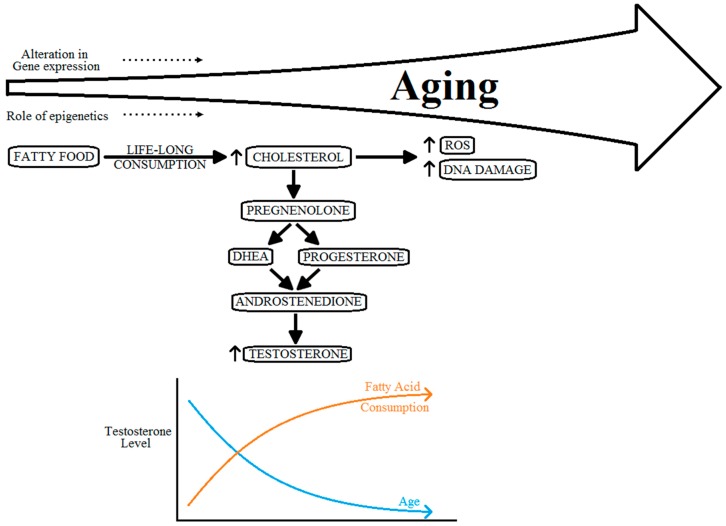
Relation between aging, cholesterol metabolism and hormonal imbalance.

Various circulating androgens, including testosterone, have important roles in the growth of the prostate gland, and PCa [64]. The specific pathway mechanism or the role of testosterone level on progression of PCa, however, is still to be well defined [64]. The level of circulating testosterone in men more than 45 years of age, across various ethnicities, shows a tendency to converge down as compared with vastly varied levels in men of age less than 30 years [52]. Many researchers have shown that higher levels of circulating testosterone do not correlate with an increased risk of PCa [65,66]. However, low levels of serum testosterone do have a direct correlation with progression of the disease to aggressive PCa [64,65,67,68]. Moreover, with a life-long consumption of fatty food substances, the levels of testosterone can also be affected by this. These absolutely contradicting effects on the levels of testosterone can also cause hormonal imbalance, as shown in Figure 4. 

These observations suggest that variation in the pattern of declining levels of free testosterone with progressing age is primarily caused by reproductive physiological variations in various populations and not because of the ethnic differences in populations [52]. It is also important to mention that the drastic depletion of testosterone levels in Caucasian populations from elevated levels in young-adulthood to comparatively much lower levels in adult-aged men causes drastic alterations in hormonal balance which may be a potential cause for higher risk of PCa [52].

Munetomo et al. (2015) have shown an increase in androgen receptor (AR) expression in the hypothalamus of rats as they age (3 v/s 24 months) [69]. Meanwhile, Pomerantz et al. (2015) have also shown extensive reprogramming of AR cistrome during prostate epithelial transformation into tumours [70]. The close correlation and co-activation between AR and telomeres have also been discussed in detail by Zhou et al. (2013) [71]. Therefore, aging related telomere shortening [72] could also have direct effects on normal functioning of the AR.

## 6. Aging, Genetic and Epigenetic Effects and Prostate Cancer

One of the other aspects linking alteration of testosterone levels, aging and PCa, and thereby, worthy of mention in this review, is alterations of DNA methylation. Ammerpohl, et al. (2013), suggested that androgens affect sexual dimorphism in humans and thereby change DNA methylation marks in the epigenome [73]. Interestingly, on one hand hypermethylation of certain genes such as *GSTP1* is well established to be consistent with the transition of PCa from intraepithelial neoplasia stage to becoming a frank carcinoma [74], and on the other, certain other studies exhibit that DNA methylation and histone modifications generally recapitulate the normal aging process [75]. Therefore, it is very difficult to specifically identify the role of aging on methylation changes, leading to and/or progression of PCa.

Drastic changes are observed in epigenetic patterns during growth and development; most of these events are biologically programmed and absolutely necessary for healthy being. However, changes in the epigenome in mature (adult) somatic tissues mirror aging-associated deleterious effects [76]. Alterations in biological processes, cellular responses, and disease states, are all parameters well-established to have an association with changes in gene expression [1]. Many microarray studies have been carried out to define the process of aging and to identify potential genes, gene expressions, and biomarkers of risk factors for many gerontological diseases [1,77]. However, aging gene expression studies have a number of complications. Two of the main issues faced by research in this area are the fact that only a few genes are identified as being differentially expressed, and also, fewer genes are found to overlap with the effect in a wide range of tissues [1,78]. Rodwell, et al. (2004), suggested that a very small proportion of transcriptional response is tissue-specific, and therefore molecular signatures of aging may overall be identified even in unrelated tissues [79]. These signatures, however, can be subject to varied interpretations rather than an active aging program [1,79]. de Magalhes, et al. (2009), were able to integrate gene expression analyses from various studies to identify genes that have a tendency to over- and/or under-express with progressing age [1]. The inflammatory response pathway is one of the most important pathways that is upregulated with aging in humans [1]. Over-expression of anti-apoptotic genes and cell-cycle regulators such as *granulin* (*GRN*), annexins, and genes playing a protective role during oxidative stress and detoxification of lipid peroxidation of end products such as *glutathione S-Transferase-1* (*GST1*) are commonly observed to be directly related to progressing age [1]. Genes that under-express with aging are not only fewer than the over-expressing genes, but are also simpler to interpret, as they are predominantly identified in the energy metabolism categories such as cholesterol metabolism [1]. Negative regulation of transcription strongly suggests that transcriptional activities decrease with aging, thus supporting the hypothesis that RNA synthesis decreases with aging [1]. Nevertheless, the total protein content of an individual may not alter with age always; therefore, decreasing mRNA may lead to the accumulation of proteins with anomalies [1].

The bulk of the mammalian genome gets transcribed to non-coding ribonucleic acids (RNAs) [80,81]. Two major groups of non-coding RNAs that play important roles as epigenetic regulators of gene expression are long non-coding RNAs (lncRNAs) [81] which are comprised of nucleotide sequences >200 bp and small non-coding RNAs which include microRNAs (miRNAs) [82], comprised of nucleotide sequences <200 bp. Certain molecular mechanisms permit lncRNAs to regulate cellular and tissue functions either by activating or suppressing gene expression [81], whereas miRNAs usually down-regulate gene expression either by mRNA degradation or by blocking off translation [82,83]. The mechanisms of action of long non-coding RNA with regards to fat metabolism, diabetes, diseases of mitochondrial dysfunction, age-associated muscle pathology, cancer, declining stress response, age-associated immune decline and age-associated neurodegeneration should also be looked into [84]. A link between miRNAs and longevity has already been demonstrated in *Caenorhabditis elegans*, thereby implicating the vital role of miRNAs in the regulation of lifespan and the overall process of aging [82]. miRNAs have a higher impact as gene modifiers compared to lncRNAs because not only can a single miRNA target multiple mRNAs, but one mRNA can also be targeted by multiple, very different miRNAs [82].

Hooten, et al. (2010), used mononuclear cells obtained from peripheral blood to estimate the expression level of miRNA in young and old populations, and identified a negative correlation with advancing age [85]. This information is very important to define the possibility of miRNA playing a crucial role in the process of aging. miRNAs have also been identified to be key players in controlling metabolic homeostasis and related diseases in individuals [86]. 

Owing to the unique expression patterns in tissues affected with cancer, miRNA profiling in PCa is a common approach adapted to diagnostic, progressive and/or therapeutic use [87]. Certain miRNAs have been identified to be associated with PCa. One such example is miR-21 [88]. miR-21 has also been identified to have increased expression in the liver biopsies of obese individuals [89]. This correlation between the role and abundant expression of miR-21 is important to understand the overall relation between obesity (which can be age-related) and PCa. Target and pathway analysis for serum age-associated miRNAs also explain the role played by miRNAs on PCa due to aging of an individual. We recently proposed that certain miRNAs can be begotten from food sources that may contribute to obesity and also to PCa [90,91]. Hooten, et al. (2013) also used TargetScan 6.2 to predict that miR-151a-3p, miR-181a-5p and miR-1248, targeted 115, 626 and 265 mRNAs respectively [92]. A number of neurological diseases and cancer, including PCa were identified to overlap among these three miRNAs thereby proving that age is a significant risk factor for certain cancers including PCa [92]. 

## 7. Discussion

The present review article targets two very important questions in the diagnosis of PCa- is this disease caused due to age-dependent immunosenescence, aging cholesterol metabolism, and androgen axis, or does failing genetics play the most crucial role in the expression and progression of PCa. The four parameters that have been followed in this review, namely changes in immunity and inflammatory response, cholesterol metabolism and obesity, effects on free testosterone level, and variations in gene expression, are all inter-linked to each other with regards to their tendency to impact expression and progression of PCa in humans with advancing age. These four parameters have isolated and combined effects on the expression and progression of PCa. 

Advancing age of patients, for example, alters the ability of certain genes to express and push towards an overall tendency to over- or under-express. The expression levels of certain genes, such as *Glutathione Transferases*, have previously been identified to have a direct relation to progressing age, and these genes in turn affect the expression of diseases such as PCa through various pathways such as inflammatory response. Interestingly, a similar chain of events pertaining to PCa can be observed when considering the effect on fat-mass and obesity-associated gene [93] initiated by progressing age. The effect of progressing age on switching on and off genes, especially on post-transcriptional gene modifiers such as lncRNAs and miRNAs are also well documented [94]. This is a major change as well, bearing in mind that miRNAs, constituting only around 1% of the entire genome have been estimated to target as many as 30% of the genes [95]. These direct our understanding of PCa towards the conclusion that it is in fact a gerontological disorder that gets aggravated with alterations in the expression of genes that govern the immune system, cellular metabolism and testosterone production. Stromal weakness with age adds to this problem, providing a platform for easy invasion of the malignant cells. 

Since these changes occur at the molecular level, it will be very interesting to see if they can be reversed using modern biological tools such as siRNA technology or clustered regularly interspaced short palindromic repeats (CRISPR)/CRISPR associated protein 9 (CRISPR/Cas9) targeted genome editing, accompanied with lifestyle changes. Therefore there is a need to be able to ablate the genes in cells so that we can understand what the loss of function would look like and to have a model where we can add back the protein in the absence of confounding local production. This situation has changed recently, however, and it is now possible to achieve very high efficiency gene targeting using the CRISPR/Cas9 technology [96,97]. As a result it is now possible to generate somatic gene knockout to produce isogenic cancer cell models that possess or lack the genes with abnormal expression and use the synthetic protein to study its mechanism of action. Even if highly targeted CRISPR/Cas work in humans requires extensive work into the future, this technology can be used in in vitro models to understand downstream pathways modified by such gene deletions. Such knowledge can be used in future targeted therapies. Until then, however, molecular techniques such as genotyping should be used in parallel with the pathophysiological details.

This brings us to the third question being addressed in this review- can diagnostic tools identify aggressive PCa from non-aggressive PCa. It is recognized that aging cannot be reversed, but, genetic tests such as association of various clinical characteristics with single nucleotide polymorphisms (SNPs) can be used for early diagnosis and personalized treatment [12,98,99]. The use of prostate-specific antigen (PSA) levels in PCa diagnosis is still controversial [100]. A strong statement by the United States Preventive Services Taskforce in 2008 and 2012 against PSA testing, as well as those of other national bodies [101,102,103], has thrown the use of PSA testing into doubt, resulting in confusion amongst patients and their health practitioners. Recent studies indicate the consequences following the withdrawal of PSA screening which has resulted in an overall increase in metastatic PCa incidence [104]. According to these authors, the relative increase in metastatic PCa incidence compared to data of 2004 was highest in the age range of 55–69 years with an increase by 92%. 

Various genetic studies with aggressive PCa have been carried out [105,106]. Such studies can pave way for early differentiation of men that are more likely to develop aggressive disease. Such information will support precautionary lifestyle changes for at risk men as well as differentiation of those that required early interventions for aggressive PCa treatments. Various genome-wide association studies (GWAS) have also been carried out to identify the possibility of aggressiveness of PCa associated with SNPs [107,108], but a well-documented database is needed to account for ethnic variations. GWAS provide the tools to identify common and low-penetrance loci of diseases, such as PCa, without prior knowledge of the location and/or function [108]. The database thereby created, will aid in identify the important SNPs and the effect of external factors, including and not limited to aging, downstream of the genes harboring the SNPs. Data sharing at this level will also be of much help for researchers to understand gene–gene, gene–environment and gene–diet interactions. Race is another risk factor especially African-American, and in almost all research carried out, it is corrected for [109], but the use of database thus created can also help researchers better understand aging process, if different between African American and Caucasian with PCa, which is not consistent even in GWAS [110], and thus help diagnose and control the progression of the disease. It will also be interesting to see if an approach targeting SNPs in genes specific to immunity and inflammatory response, cholesterol metabolism and obesity, and testosterone metabolism is carried out to check for association with progression of PCa in patients with and without the aggressive form of the disease. 

## Abbreviations

The following abbreviations (mentioned alphabetically) are used in this manuscript:

*ANXA1*: *Annexin A1*
CRISPR: clustered regularly interspaced short palindromic repeats
CRISPR/Cas9: CRISPR associated protein 9
DHEA: dehydroepiandrosterone
*GDF15*: *Growth differentiation factor 15*
*GRN*: G*ranulin*
*GST1*: *Glutathione S-Transferase-1*
*GSTP1*: *Glutathione S-Transferase P*
GWAS: genome-wide association studies
HDL: high-density lipoprotein
HFDs: high fat diets
*IL10*: *Interleukin -10*
*IL1RN*: *Interleukin 1 receptor antagonist*
*IL8*: *Interleukin -8*
LDL: low-density lipoprotein
lncRNAs: long non-coding RNAs
*MIC-1*: *Macrophage inhibitory cytokine-1*
miRNAs: microRNAs
*MSR1*: Macrophage *Scavenger Receptor 1*
PCa: prostate cancer
PSA: prostate-specific antigen
RNAs: ribonucleic acids
*RNASEL*: *RNase L*
ROS: reactive oxygen species
SNPs: single nucleotide polymorphisms
*TLR4*: *Toll-like receptor 4*
